# Supplementary material for: Entorhinal grid-like codes and time-locked network dynamics track others navigating through space
Source: Nat Commun. 2023 Jan 31;14:231. doi: 10.1038/s41467-023-35819-3 (PMC9889810; doi:10.1038/s41467-023-35819-3)
Supplement: Supplementary file 1 — Supplementary Information [file 41467_2023_35819_MOESM1_ESM.pdf]

Supplementary Information

Increased activation in the right posterior parietal cortex during observation is associated with better performance

Since participants generally performed best when re-tracing the first path segment of a given trajectory (**Supplementary Fig. S1**), we reasoned that good performance might rather be reflected by a smaller distance error on the second and third path segments (i.e., the cumulative distance $error_{S2+S3}$). We thus repeated our analysis, regressing the cumulative distance $error_{S2+S3}$ against voxel-wise brain activity. Results were virtually identical, again showing increased activation in the right posterior parietal cortex ($x = 44$, $y = -78$, $z = 24$, z-value = 4.5, 226 voxels) during observation that negatively scaled with the individual cumulative distance error across path segments 2-3 [linear regression, $N = 58$, contrast observation > baseline, cumulative distance $error_{S2+S3}$ added as a covariate of interest; $p < 0.05$ family-wise-error (FWE) corrected at cluster level using a cluster-defining threshold of $p < 0.001$, cluster size = 80 voxels].

Entorhinal grid-like codes when observing the demonstrator's paths

Our main results of significant grid-like codes during observation remained stable when we delineated the entorhinal cortex following a manual segmentation protocol (**Methods**). In brief, we found significant grid-like codes in the entorhinal cortex as participants were observing the demonstrator's paths. This effect was only present for the 6-fold symmetrical model [one-sample t-test, $N = 51$ (full data set, no exclusions); $t(50) = 1.9$, Cohen's $d = 0.27$, 95% confidence interval (CI) = [-0.01, 0.18], $p_{\text{one-tailed}} = 0.032$, although not surviving correction for multiple comparisons; **Supplementary Fig. S3a;** results for this and all following analyses of this section remained stable when using the full data set, see also **Supplementary Table S2**]. Different symmetrical models such as 5- or 7-fold signal periodicities did not yield significant results [5-fold: $N = 47$ (excluding 4 outliers), $p_{\text{one-tailed}} = 0.102$; 7-fold: $N = 46$ (excluding 5 outliers), $p_{\text{one-tailed}} = 0.178$; **Supplementary Fig. S3a**].

Significant entorhinal grid-like codes during observation when using a different cross-validation regime

Our initial cross-validation regime involved estimating the putative grid orientation on 11 of 12 trials (GLM1) and testing the signal modulation on the remaining three path segments of the held-out trial (GLM2). To assure that the parametric modulation of the held-out data could be reliably estimated, we repeated the main analysis and used a modified, less nested cross-validation regime by splitting each task run into four data parts [i.e., estimating grid orientations on three fourths (27 path segments) and testing on the remaining (9 path segments)]. Results remained stable, showing significantly increased grid-like codes in the entorhinal cortex (defined through automated segmentation using ASHS, **Methods**) during observation [6-fold symmetrical model, mean grid magnitude (arbitrary units, arb. units) ± s.e.m., 0.077 ± 0.04, one-sample t-test, $N = 45$ (excluding 4 outliers); $t(44) = 2.013$, Cohen's $d = 0.3$, 95% CI = [0, 0.15], $p_{\text{one-tailed}} = 0.025$].

Entorhinal grid-like codes during observation partly generalize across runs

Our main goal was to probe grid-like codes during observation. It has been shown that several factors can affect the fMRI signal over longer time periods (e.g., within one fMRI session) which makes it difficult to compare between separate task runs (see also the **Methods** section for a mode in-depth discussion of this aspect). In brief, these factors include substantial signal drifts of fMRI-based activation patterns over time[1] as well as potential imprecisions in the spatial co-registration between different task runs. Most

importantly, grid orientations can "remap" even in the same spatial environment[2,3] and this could be triggered by the end of one task run and the beginning of the next (indicating an "event boundary"). The precise reason for fluctuations in grid orientations are currently unclear. For these reasons, we explicitly opted for cross-validation *within* task runs.

Nevertheless, to test whether entorhinal grid-like codes during observation were stable over longer time periods we repeated the main analysis and used a cross-validation regime that estimated and tested observation-related grid orientations in the entorhinal cortex (automatic segmentation, identical to main analysis) across separate task runs (there were four separate runs during the modified navigation task). We estimated grid orientations on even runs (i.e., runs 2 and 4, mean grid orientations estimated across both) and tested grid orientations on the remaining (i.e., runs 1 and 3). Results showed significantly increased grid-like codes during run 2 [mean grid magnitude (arbitrary units, arb. units) ± s.e.m., 0.03 ± 0.01; one-sample *t*-test, $N = 48$ (excluding one formal outlier); $t(46) = 2.39$, $d = 0.36$, 95% CI = [0.005, 0.6], $p_{\text{one-tailed}} = 0.011$, Bonferroni-corrected for multiple comparisons using a threshold of $\alpha_{\text{Bonferroni}} = 0.05/2 = 0.025$) but not during run 4 ($N = 48$ (excluding one formal outlier), $p_{\text{one-tailed}} = 0.315$). Thus, entorhinal grid-like codes partly generalized across runs during observation, speaking for a certain degree of stability of grid orientations over longer time periods.

The partial generalization does not come surprising to us. Our modified navigation task is novel and entirely different compared to other tasks that are typically used to probe entorhinal grid-like codes during navigation (such as the object-location task)[4,5]. Participants observed short paths on each trial (consisting of three path segments each) and re-traced them thereafter. The switch between observation/navigation periods and the change from one path to the next could have enforced the perception of "event boundaries", potentially contributing to "remapping" of grid orientations and generally lower temporal stability. Indeed, while we found no significant difference in the temporal stability of grid orientations between the observation/navigation periods, temporal stability within runs was generally low (**Supplementary Fig. 8**). We expect this "remapping" to also be the case as participants complete one task run and start the next run after a short break, which could have contributed to the abovementioned results.

Grid-like codes during observation appear left lateralized

Since the grid-like modulation of the entorhinal signal was reported to be right-lateralized in previous navigation studies using virtual reality[4,6], we repeated our analyses but separately tested for entorhinal grid-like codes in the left and right hemisphere (ROIs defined through automated segmentation using ASHS). We used the cross-validation scheme reported above (data was partitioned into four parts for estimating and testing individual grid orientations). Participants with less than 5 voxels within the ROI mask were excluded from the analyses which yielded a final sample of 38 and 37 participants for the left (mean ± s.d., 13 ± 7 voxels) and right (14 ± 7 voxels) hemisphere, respectively.

Findings revealed significant grid-like codes in the left [6-fold symmetrical model, mean grid magnitude (arbitrary units, arb. units) ± s.e.m., 0.078 ± 0.03, one-sample *t*-test, $N = 37$ (excluding 1 outlier); $t(36) = 2.421$, Cohen's $d = 0.4$, 95% CI = [0.01, 0.14], $p_{\text{one-tailed}} = 0.021$] but not in the right entorhinal cortex during observation [6-fold symmetrical model, mean grid magnitude (arbitrary units, arb. units) ± s.e.m., -0.025 ± 0.05, one-sample *t*-test, $N = 37$ (full sample); $p_{\text{one-tailed}} = 0.339$].

Negative grid-like codes in V1: different cross-validation regime

We further investigated the result of significantly negative grid-like codes in primary visual cortex (V1) during observation. One reason for this result could be the small number of path segments in the test set using our initial 12-fold cross-validation regime (grid orientations were iteratively estimated on 11

trials/33 path segments and tested on 1 trial/3 path segments), whereby observing/moving in single directions could have driven the result (e.g., a strong response in a specific direction compared to smaller responses in other directions). We thus repeated our analysis with a different cross-validation regime (reported above, splitting the data into four parts for estimating/testing grid orientations). However, results remained unchanged and once more revealed significantly negative grid-like coding in V1 during observation [6-fold symmetrical model, mean grid magnitude (arbitrary units, arb. units) ± s.e.m., -0.82 ± 0.11, one-sample t-test, N = 55 (excluding 1 outlier); $t(54) = -7.25$, Cohen's $d = -0.98$, 95% CI = [-1.04, -0.59], $p_{\text{one-tailed}} < 0.0001$].

Negative grid-like codes in V1: increased activity when observing misaligned paths

Results of negative grid-like coding emerged as surprising side-findings in our work, and from what we understand, also had emerged unexpectedly in previous work[7]. A significantly negative 6-fold symmetrical modulation of the fMRI signal in V1 could indicate increased grid magnitudes for directions that are rotated 30°, resulting in increased signal opposite to the estimated grid orientation.

To determine whether this was the case also in our data set, we repeated the main grid analysis but now modelled V1 activation levels with separate regressors for the different movement directions that participants observed. Voxel-wise grid orientations were estimated identically to our initial analysis (using a 12-fold cross-validation regime). Importantly, GLM2 was now used to contrast directions aligned (0 modulo 60°) versus misaligned (0 modulo 30°) relative to the estimated grid orientation. Thus, V1 activation levels were modelled with separate regressors for the different movement directions that participants observed (instead of parametrically modulating each path segment using the difference between each segment's translational direction and the participant's mean grid orientation, as we had done previously, see **Methods**). Next, we averaged the obtained grid magnitudes across cross-validation folds, runs, and voxels within the V1 ROI. We plotted the parameter estimates (arbitrary units, arb. units) for each directional bin, and also assessed the averaged signal change across all aligned and misaligned directional bins.

We found that V1 activation levels appeared increased for observed directions that were misaligned with the individual grid orientation (0 modulo 30°) compared to aligned directions (0 modulo 60°). This effect was not driven by single directional bins but appeared relatively consistent across the range of misaligned directions (**Supplementary Fig. S5a**). Next, we assessed the average activation levels across all aligned and misaligned directions. Activation for misaligned directions (mean ± s.e.m., 0.84 ± 0.22) was significantly higher than for aligned directions (0.4 ± 0.2; paired-sample t-test, N = 58 (full sample), $t(57) = -2.48$, $p_{\text{two-tailed}} = 0.016$; **Supplementary Fig. S5b**). Our effects therefore appear related to decreased V1 activity when participants observed the demonstrator walking aligned with their individual grid orientation.

Multiple explanations of how grid cell firing activity relates to macroscopic (fMRI-based) signals exist (for a review, see[8]). For example, increased grid-like coding might be related to the crossing of multiple firing fields (leading to an elevated fMRI signal). In contrast to this, different results can be obtained if repetition suppression mechanisms are at play (for a review, see[9]). Grid cells would repeatedly fire during aligned movement and this repetition would cause a suppression of neuronal firing (leading to a decreased fMRI signal), given that inhibitory mechanisms appear vital for grid-like coding[10]. Thus, it is possible that the decreased V1 activity for misaligned > aligned directions could represent hexadirectional signal modulation in the form of neural adaption. Also, grid-like neural adaption effects in V1 could be related to grid cells in the visual system (although it is currently unclear whether grid cells exist in V1, but see a recent preprint reporting on grid cells in V2[11]) or to effects that are caused by grid cell activity upstream (from the entorhinal cortex). At present, however, we do not know which factors drive this effect and cannot provide a firm interpretation of why negative grid-like coding in V1 appears associated with

tracking others navigating through space. We encourage future research to elucidate this finding, as well as the relationship between grid-like codes in the medial temporal lobe and visual systems.

Entorhinal grid-like codes during observation are stronger at lower performance

Brain-behavior relationship is unrelated to the length of the observed paths

We performed additional analysis to clarify whether our finding of increased entorhinal grid-like codes during observation at lower performance might depend on path differences between high- vs. low-performance trials (i.e., trials with a smaller vs. larger cumulative distance error, respectively). Paths during low-performance trials might have crossed the center of the environment more often, leading to a longer average path length and potentially contributing to increased grid-like coding.

We tested whether this was the case and calculated the average path length of high- vs. low-performance trials (defined as trials with <= 20 vm or > 20 vm cumulative distance error). There was no significant difference between the average path length of high- vs. low-performance trials [paired-sample *t*-test, N = 59 (full sample), $p_{\text{two-tailed}} = 0.095$; high-performance trials: 110.2 ± 0.03 vm, low-performance trials: 105.3 ± 0.38 vm]. Also, there was no significant association between the magnitude of entorhinal grid-like codes during observation and the average path length observed across participants [N = 46 (3 outliers excluded, same sample as in original analysis), $p_{\text{two-tailed}} = 0.071$]. We conclude that the brain-behavior association does not stem from differences in path length between high- and low-performance trials and added this analysis to the manuscript.

Brain-behavior relationship remains stable when using a normalized accuracy measure

We repeated the correlation analysis with a newly-defined performance measure, taking into account that chance performance for re-tracing a given path correctly differed for different endpoints along the circumference of the movement area[6].

In our modified navigation task, participants were asked to re-trace the demonstrator's paths as accurately as possible, moving from a starting point towards specific endpoints along the circumference of the circular movement area (that is, participants were not able to stop and turn within the movement area). A single path consisted of three path segments. For each path segment that a participant re-traced after having observed the demonstrator, we calculated the probability distribution of all possible Euclidean distance errors. In other words, we simulated all possible endpoints that a participant could have navigated towards, spanning the entire circumference of the movement arena in 1°-degree steps (**Supplementary Fig. S6a**). We then calculated the Euclidean distance between the correct endpoint and all other possible points, including the endpoint that the participant actually navigated towards. Based on this probability distribution, we determined the percentile rank of the actual error distance of a given path segment (i.e., the endpoint that a given participant actually navigated towards) relative to all possible error distances and determined its accuracy score (1-percentile/100; **Supplementary Fig. S6b**). Higher values thus indicated that participants re-traced the observed path segments more precisely. Altogether, this yielded accuracy scores that were normalized with respect to differences in the probability of obtaining a correct response at different areas of the circular environment.

The mean accuracy across participants was ~86%. Next, we tested whether this different method for assessing individual performance affected our main result regarding the brain-behavior relationship of increased grid-like magnitudes at lower individual performance across participants and repeated the correlation analysis. Results remained virtually unchanged, once again showing increased grid-like codes

in participants with lower overall accuracy (N = 47, same sample as for main analysis: $r_{Pearson}$ = -0.43, 95% CI = [-0.64, -0.16], $p_{\text{two-tailed}}$ = 0.003; **Supplementary Fig. S6c**).

No significant entorhinal grid-like codes when re-tracing the demonstrator's paths

We did not find significant grid-like codes in the entorhinal cortex, also not when manually delineating the region [N = 45 (excluding 6 outliers), $p_{\text{one-tailed}}$ = 0.323; **Supplementary Fig. S3b**; results for this and all following analyses of this section remained stable when using the full data set of N = 49 and when using a robust test framework, **Supplementary Table S3**], or when testing for grid-like codes in the hippocampus [N = 52 (excluding 6 outliers), $p_{\text{one-tailed}}$ = 0.34], the parahippocampal cortex [N = 56 (excluding 2 outliers), $p_{\text{one-tailed}}$ = 0.087], or V1 [N = 52 (excluding 6 outliers), $p_{\text{one-tailed}}$ = 0.222; **Supplementary Fig. S3b**]. However, we found significantly increased grid magnitudes in the anterior thalamus which was present when applying a 6-fold symmetrical model to the data [N = 54 (excluding 4 outliers), $t(53)$ = 2.186, d = 0.3, 95% CI = [0.02, 0.48], $p_{\text{one-tailed}}$ = 0.016, although not surviving correction for multiple comparisons; **Supplementary Fig. S3b**].

No significant grid-like codes during navigation when using a reduced data set

Additionally, we speculated that the lack of grid-like codes when re-tracing the demonstrator's paths might have been caused by the specifics our task design. Participants were placed directly behind the demonstrator's starting position during observation (note that the starting position randomly varied from trial-to-trial). This might have prompted participants to adopt an ego- rather than allocentric reference frame, especially during the first path segment (the remaining path segments varied randomly relative to the participant's initial viewpoint). If this was the case, we would have expected decreased grid-like codes in the entorhinal cortex when traversing path segments that were decoupled from the initial viewpoint (i.e., path segments 2-3). We thus repeated the above analysis and tested for grid-like codes during navigation when re-tracing the initial vs. the remaining path segments (i.e., collapsing across path segments 2-3; **Methods**). Results did not show significant grid-like codes in the entorhinal cortex when participants re-traced the initial path segment [one-sample t-test; N = 42 (excluding 7 outliers), $p_{\text{one-tailed}}$ = 0.09; N = 49 (full sample), $p_{\text{one-tailed}}$ = 0.156], and also not when they re-traced segments 2-3 [one-sample t-test; N = 38 (excluding 11 outliers), $p_{\text{one-tailed}}$ = 0.125; N = 49 (full sample), $p_{\text{one-tailed}}$ = 0.156], confirming our initial findings (however, please note that these results are based on a reduced data set which might have contributed to the lack of significant findings).

No significant grid-like codes during navigation when using a different cross-validation regime

As mentioned previously, our initial cross-validation regime involved estimating the putative grid orientation on 11 of 12 trials (GLM1) and testing the signal modulation on the remaining three path segments of the held-out trial (GLM2). Such heavily nested cross-validation might negatively affect the reliable estimation of the parametric modulation in the held-out data set. We thus repeated the main analysis and used a modified, less nested cross-validation regime by splitting each task run into four data parts (see above). Results remained stable, showing no significant grid-like codes in the entorhinal cortex (defined through automated segmentation using ASHS, **Methods**) during navigation [6-fold symmetrical model, one-sample t-test, N = 46 (excluding 3 outliers), $p_{\text{one-tailed}}$ = 0.247].

Grid-like codes during navigation appear right lateralized

Following our approach above, we repeated the analyses but separately tested for entorhinal grid-like codes in the left and right hemisphere (ROIs defined through automated segmentation using ASHS; N =

38 (left) and 37 (right); mean ± s.d.; left ROI: 13 ± 7 voxels, right ROI: 14 ± 7 voxels; 4-fold cross-validation regime). We found significantly negative grid-like codes in the left [6-fold symmetrical model, mean grid magnitude (arbitrary units, arb. units) ± s.e.m., -0.071 ± 0.04, one-sample *t*-test, *N* = 37 (excluding 1 outlier); $t(36) = -1.817$, Cohen's $d = -0.29$, 95% CI = [-0.15, 0.01], $p_{\text{one-tailed}} = 0.039$] and numerically increased (but not significant) grid-like codes in the right entorhinal cortex during navigation [6-fold symmetrical model, mean grid magnitude (arbitrary units, arb. units) ± s.e.m., 0.085 ± 0.05, one-sample *t*-test, *N* = 37 (full sample); $p_{\text{one-tailed}} = 0.06$].

Results indicating negative grid-like coding in the visual (or entorhinal) cortex emerged as surprising side-findings not only from our analyses, but had already been reported as equally unexpected findings in previous work[7]. Additionally, there is an ongoing debate in the field on how grid cell firing relates to macroscopic (fMRI-based) signals[8]. Thus, we are generally unsure about what could cause a switch from increased (see above) to decreased grid-like codes in the left entorhinal cortex during observation and navigation, respectively. One possibility could be that increased grid-like codes during observation are suppressed during subsequent navigation, as could be explained by a neural adaption mechanism. However, we directly tested whether neural adaption could have led to a decrease in the overall entorhinal cortex signal during navigation and this was not the case (see results below). We encourage future studies to scrutinize the finding of negative grid-like codes and to determine how fMRI-based grid-like codes relate to underlying grid cell firing.

Grid orientations during observation did not match those during navigation

We further explored whether grid orientations during observation periods served as spatial reference frames when participants re-traced the demonstrator's paths during navigation. If this was the case, we would expect to find matching grid orientations between the two conditions, leading to significantly increased grid magnitudes when testing grid orientations obtained from observation- on navigation periods. We repeated the above analysis but estimated individual grid orientations based on all paths during the 12 observation periods of each task run, testing them on all paths during the 12 navigation periods of each task run (**Supplementary Fig. S7a**). Results did not yield significant grid magnitudes in the entorhinal cortex when testing grid orientations obtained from observation- on navigation periods [one-sample *t*-test, *N* = 45 (excluding 4 outliers), $p_{\text{one-tailed}} = 0.206$; *N* = 49 (full sample), $p_{\text{one-tailed}} = 0.156$; **Supplementary Fig. S7a**] or vice versa [i.e., grid orientations obtained from navigation periods did not match those during observation*;* *N* = 45 (excluding 4 outliers), $p_{\text{one-tailed}} = 0.46$; *N* = 49 (full sample), $p_{\text{one-tailed}} = 0.494$; **Supplementary Fig. S7a**]. Individual grid orientations per condition, as well as the difference in grid orientation angles between the conditions are shown in **Supplementary Fig. S7b-d**.

No effect of neural adaption during navigation

One possible reason for the lack of significant entorhinal grid-like codes during navigation could be neural adaption (or "repetition suppression") during navigation. In the current task, observation periods were followed by navigation periods and neural adaption might thus indicate an association (or similarity) between the periods, perhaps in the form of a "memory trace" (for a review, see[9]). To be able to compare between navigation periods with high/low similarity to the previous observation period, we took into account individual performance: we expected that the observation-navigation similarity should be stronger for trials during which participants performed well (compared to trials during which participants performed less well; i.e., smaller vs. larger cumulative distance error, respectively). In other words, during high-performance trials, participants should have encoded the path trajectory well during observation, indicated by a stronger neural adaption during the subsequent navigation period.

We created a model that was similar to the initial whole-brain univariate fMRI analysis (independent of grid-like coding) but that incorporated a different regressor structure. To briefly explain, we used a Generalized Linear Model (GLM) to model the BOLD response during the modified navigation task with task regressors that were time-locked to the onsets of the respective events (cue + feedback periods, high-performance observation, low-performance observation, high-performance navigation, low-performance navigation). High- and low-performance trials were defined according to the feedback threshold employed in the modified navigation task (<= 20 vm or > 20 vm cumulative distance error). Participants generally performed well [they showed significantly more high- (mean ± standard error of the mean, s.e.m., 7.59 ± 0.29 trials per run) compared to low-performance trials (4.41 ± 0.29 trials per run); N = 44, paired-sample t-test, $t(43)$ = 5.46, Cohen's d = 0.82, 95% confidence interval (CI) = [1.99, 4.34], $p_{\text{two-tailed}} <$ 0.0001]. Note that this analysis did not include 14 participants who did not display any low-performance trials.

All task events were estimated as boxcar functions of specific durations and were convolved with the SPM default canonical hemodynamic response function (HRF). Cue and feedback periods were modelled with a duration of 2 and 1 seconds, respectively, and were collapsed within one regressor. The duration of observation and navigation periods varied depending on the path length and the participants' behavior and was defined through the on- and offsets of the VR environment on the computer screen (ranging between 18-40 seconds). This included events such as orientation adjustments (rotations), walked path segments (translation periods), and time periods during which no movement occurred (short standing periods in-between). To account for noise due to head movement, we included the six realignment parameters, their first derivatives, and the squared first derivatives into the design matrix. A high-pass filter with a cutoff at 128 s was applied. The four runs of the modified navigation task were combined into one first-level model and contrasts collapsing across the different task runs were created (high-/low-performance navigation > implicit baseline). Next, we took the anatomical boundaries of the bilateral posterior-medial entorhinal cortex[12], extracted the average activation levels during high- and low-performance navigation periods and compared them.

Results did not reveal a significant difference in entorhinal cortex activation between high- (0.41 ± 0.12 parameter estimates, arbitrary units, arb. units) and low-performance trials (0.33 ± 0.08) as participants re-traced the demonstrator's path trajectories (paired-sample t-test, N = 44 (full sample), $p_{\text{two-tailed}}$ = 0.456; results did not change when excluding 2 outliers, N = 42, $p_{\text{two-tailed}}$ = 0.229; **Supplementary Fig. S9a**). Based on this analysis approach, we did not find strong evidence for neural adaption during navigation.

Additional analysis of entorhinal cortex activation

Next, we drew upon our initial analysis of whole-brain univariate activation levels (independent of grid-like coding, see also **Fig. 2**) and assessed aspects of neural adaption as well as the relationship between entorhinal activation levels and grid-like codes (note that we previously had applied whole-brain, cluster-based correction for multiple comparisons and thus might have missed any significant results pertaining to the relatively small entorhinal cortex region).

First, we performed an analysis similar to the neural adaption analysis above. To avoid any artificial segregation into high- vs. low-performance trials, we extracted the average activation levels during navigation from the entorhinal cortex (same anatomical mask as above) and measured their cross-participant correlation with individual performance (vm, averaged across all three points of a path trajectory; similar to the whole-brain linear regression results presented in **Fig. 2d**). In the case of neural adaption, we would expect decreased activation at higher individual performance (in line with our finding of decreased grid-like coding at higher individual performance). Second, we determined whether

activation levels during observation/navigation differed in the entorhinal cortex (generally lower activation during navigation could explain our null-finding regarding grid-like codes).

There was no significant correlation between navigation-related activation levels in the entorhinal cortex and individual performance (*N* = 58 (full sample), Pearson correlation, $p_{\text{two-tailed}} = 0.755$; **Supplementary Fig. S9b**), confirming the results from the neural adaption analysis above. Next, we found that entorhinal activation levels were not significantly different between observation and navigation periods (*N* = 58 (full sample), paired-sample *t*-test, $p_{\text{two-tailed}} = 0.831$; **Supplementary Fig. S9c**). Hence, our finding regarding no significant grid-like codes during navigation appears unrelated to general activation differences between the conditions.

No effect of eye movements on grid-like codes

We repeated our initial grid analysis but instead modelled eye gaze directions (i.e., the angle between successive saccades with respect to an arbitrary reference point on the computer screen) instead of movement trajectories in VR-space (note that eye-tracking data was available only for a subset of 47 participants; of these, grid code analyses were possible for *N* = 37 participants that had a sufficient amount of voxels in the entorhinal cortex mask; **Methods**). Each task run was partitioned into data halves to independently estimate and test grid orientations (i.e., individual grid orientations were estimated on all saccades during trials 1-6 and were tested on all saccades during trials 7-12). As above, grid orientations were assessed separately for observation and navigation periods.

Results showed no significant increase in entorhinal grid magnitudes when testing for a 6-fold symmetrical model during observation periods [separate one-sample *t*-tests; *N* = 33 (excluding 4 outliers), $p_{\text{one-tailed}} = 0.433$; *N* = 37 (full sample), $p_{\text{one-tailed}} = 0.449$]. However, the distance viewed across the different directional bins appeared biased [i.e., participants made more or longer saccades along directional bins 1 and 6 which covered horizontal eye movements between the left/right borders of the observed movement area; repeated-measures ANOVA, *N* = 33, significant main effect of directional bin, $F(5,186) = 30.1$, $\eta^2 = 0.43$, 95% CI = [245.9, 266.5], $p_{\text{two-tailed}} < 0.0001$; **Supplementary Fig. S8c**]. Since this could have distorted the estimation of grid orientations, we repeated the above analysis and randomly selected saccades to match the average, individual distance viewed along directional bins 2 to 5. Again, results showed no significant saccade-related grid-like codes in the entorhinal cortex during observation [*N* = 37 (full sample, no outliers), $p_{\text{one-tailed}} = 0.35$; **Supplementary Fig. S8c**], and also not during navigation periods [*N* = 35 (excluding two outliers), $p_{\text{one-tailed}} = 0.096$; *N* = 37 (full sample), $p_{\text{one-tailed}} = 0.027$; **Supplementary Fig. S8c**]. This suggests that our finding of significant grid-like codes in the entorhinal cortex when observing the demonstrator's paths (**Fig. 3d**) was primarily based on spatial information rather than the direction of saccadic eye movements.

Participants followed the demonstrator with their eye gaze during observation

To verify that participants actually followed the demonstrator with their eye gaze during observation, we demarcated rectangular areas-of-interest (AOIs) that were defined by the two-dimensional coordinates of a given path segment on the computer screen (i.e., its starting and end points) as well as by the demonstrator's height on the screen (thus, defining the area on the screen within which the demonstrator was moving). We then calculated the percentage of eye movements that fell within the AOI boundaries. We reasoned that participants would produce slower (smooth pursuit) as well as faster (saccadic) eye movements to track the demonstrator and hence based calculations on the raw data that was cleaned from eye blinks (i.e., data was not restricted to saccades only, as was the case for the abovementioned saccade-based grid code analysis).

Indeed, the majority of eye movements were located within AOIs, emphasizing that participants' viewing behavior was related to the observation of the demonstrator [N = 47 (full sample for which eye tracking data was available), percentage of eye movements detected within AOI per path segment, mean ± s.e.m., first path segment: 80.1 ± 2.41 %, second path segment: 77.11 ± 1.9 %, third path segment: 73.21 ± 1.68 %]. There was no difference in the amount of AOI-related eye movements across the three path segments [one-way ANOVA, N = 47, no significant main effect of path segment, $p_{\text{two-tailed}} = 0.057$].

Reported strategy use during the modified navigation task

After completing the MRI experiment, participants were interviewed regarding their individual strategies that they had adopted during observation periods ("Briefly describe in your own words how you oriented yourself within the virtual reality environment during observation/navigation."). Of all 58 participants, 16 (27.6%) reported that they paid attention to the landmarks while tracking the avatar and during self-navigation (strategy "landmark"), 30 (51.7%) reported that they paid attention to the landmarks as well as to the magnitude of the movement angles in-between subsequent path segments (strategy "landmark + angle"), 6 (10.3%) reported that they paid attention to the landmarks as well as to the duration of the rotation when the avatar was adjusting its orientation in-between path segments (strategy "landmark + rotation"), 4 (6.9%) reported that they paid attention to the landmarks and tried to imagine themselves in the demonstrator's perspective during observation (strategy "landmark + demonstrator perspective"), and 2 (3.4%) reported creating a "mental map", trying to capture the general layout of the entire environment as good as possible (strategy "mental map")[1].

It was previously shown that entorhinal grid-like codes could be detected as participants imagined movement through space while they remained stationary[7,13]. It is possible that participants were imaging their own movements while observing the demonstrator and that this mental navigation caused elevated grid-like codes during observation. From the 47 participants that were included in the main grid analysis, only one person reported to have imagined the demonstrator's perspective (the remaining three participants were excluded from this analysis because of outlier values or because of insufficient data within the entorhinal cortex ROI). We excluded this participant from the sample and repeated the main analysis (see **Results** and **Methods** section; 12-fold cross validation to detect grid-like codes during observation periods, automatic segmentation of the entorhinal cortex using ASHS). Results remained unchanged and once more confirmed our finding of entorhinal grid-like codes during observation [6-fold symmetrical model, mean grid magnitude (arbitrary units, arb. units) ± s.e.m., 0.124 ± 0.05, one-sample t-test, N = 45 (one participant excluded due to strategy, one outlier excluded), $t(44) = 2.257$, $d = 0.34$, 95% CI = [0.01, 0.24], $p_{\text{one-tailed}} = 0.015$].

[1] To clarify, we assume that the majority of participants formed a "mental map", that is, a sense of the general layout of the environment as it would have been very difficult to successfully perform the task otherwise. Unfortunately, we do not have more detailed data available on these participants as no follow-up questions were asked about how these participants actually created their "mental maps".

Supplementary Figures

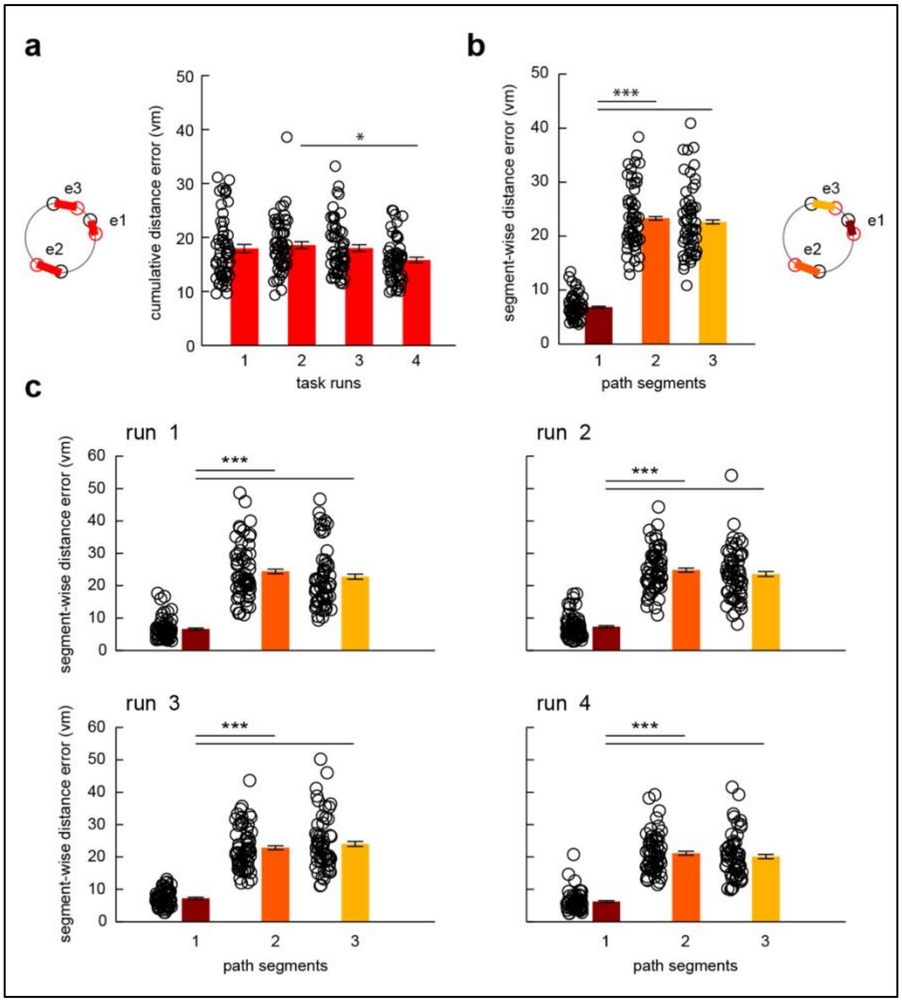

Fig. S1: Navigation performance. (a) Performance was quantified as cumulative distance error in virtual meters (vm) averaged across all three path errors [e1-e3; showing the difference between the demonstrator's (black) and participant's (red) path segment endpoints]. The cumulative distance error during the second run was higher compared to the last run [one-way analysis of variance (ANOVA), N = 58, main effect of run, $F(3,227) = 3.53$, $\eta^2 = 0.04$, 95% CI = [0, 0.1], $p_{\text{two-tailed}} = 0.016$; post-hoc pair-wise comparison run 2 vs. 4, $t(227) = 3.02$, $d = 0.62$, 95% CI = [-5.1, -0.4], $p_{\text{two-tailed}} = 0.015$; all other post-hoc comparisons $p_{\text{two-tailed}} > 0.05$]. **(b)** Across runs, participants performed best when re-tracing the first path segment [one-way ANOVA, N = 58, main effect of path segment, $F(2,171) = 190.7$, $\eta^2 = 0.69$, 95% CI = [0.63, 0.74], $p_{\text{two-tailed}} < 0.0001$; pair-wise comparisons: first vs. second segment, $t(171) = -17.24$, $d = -2.96$, 95% CI = [14.2, 18.7], $p_{\text{two-tailed}} < 0.0001$, first vs. third segment, $t(171) = -16.57$, $d = -2.91$, 95% CI = [13.5, 18], $p_{\text{two-tailed}} < 0.0001$, second vs. third segment, $p_{\text{two-tailed}} = 0.784$]. **(c)** Segment-wise distance errors per run (separate one-way ANOVAs, two-tailed, N = 58). * $p < 0.05$; *** $p < 0.0001$; error bars reflect the standard error of the mean, s.e.m. Source data are provided as a Source Data file.

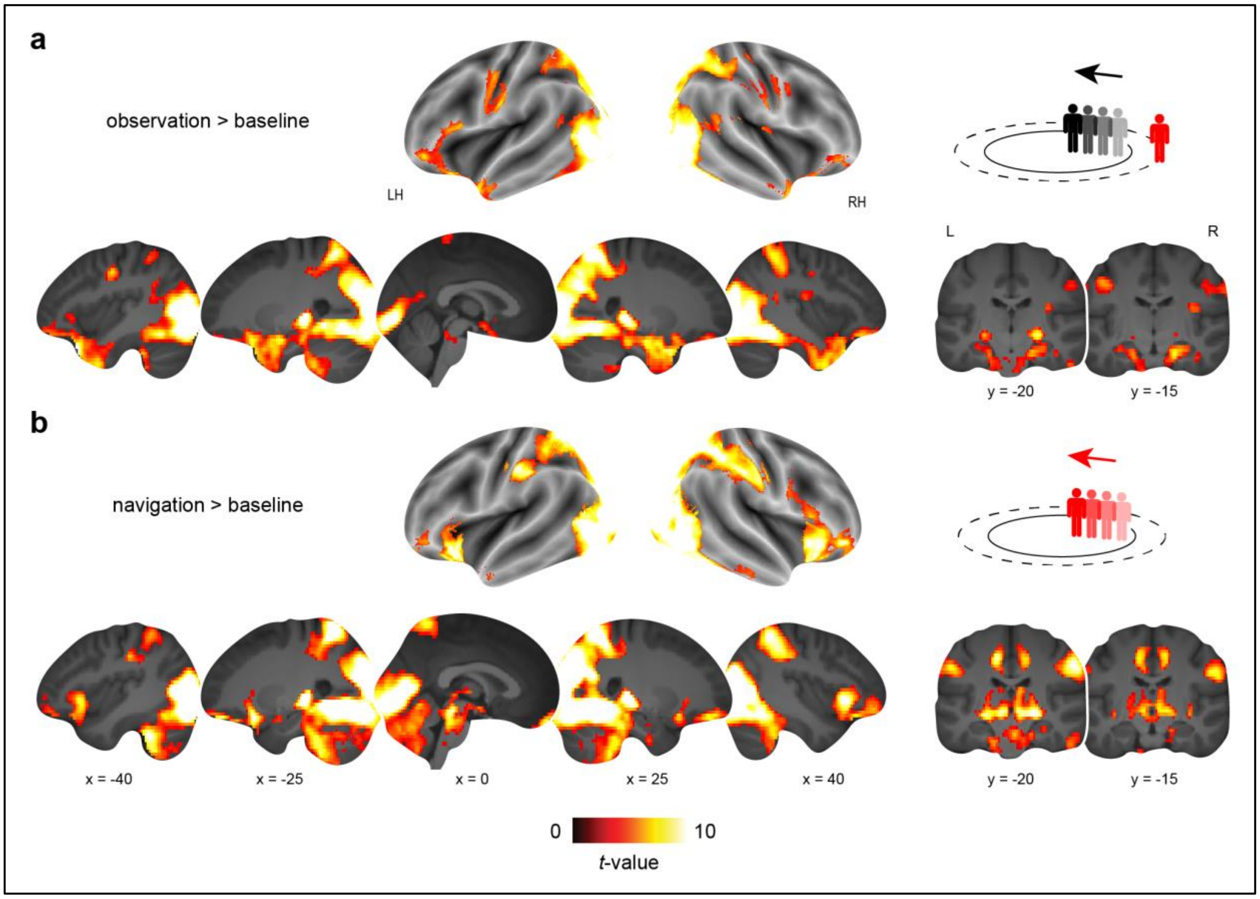

Fig. S2. Brain activation profiles during observation and navigation. Brain activation **(a)** during observation and **(b)** navigation, each compared to the implicit (fixation) baseline (separate one-sample *t*-tests, $N = 58$; **Supplementary Table S1**), plotted onto brain slices of the average structural image. All results are shown at $p < 0.05$ FWE-corrected at cluster level (cluster-defining threshold of $p < 0.001$). Source data are provided as a Source Data file.

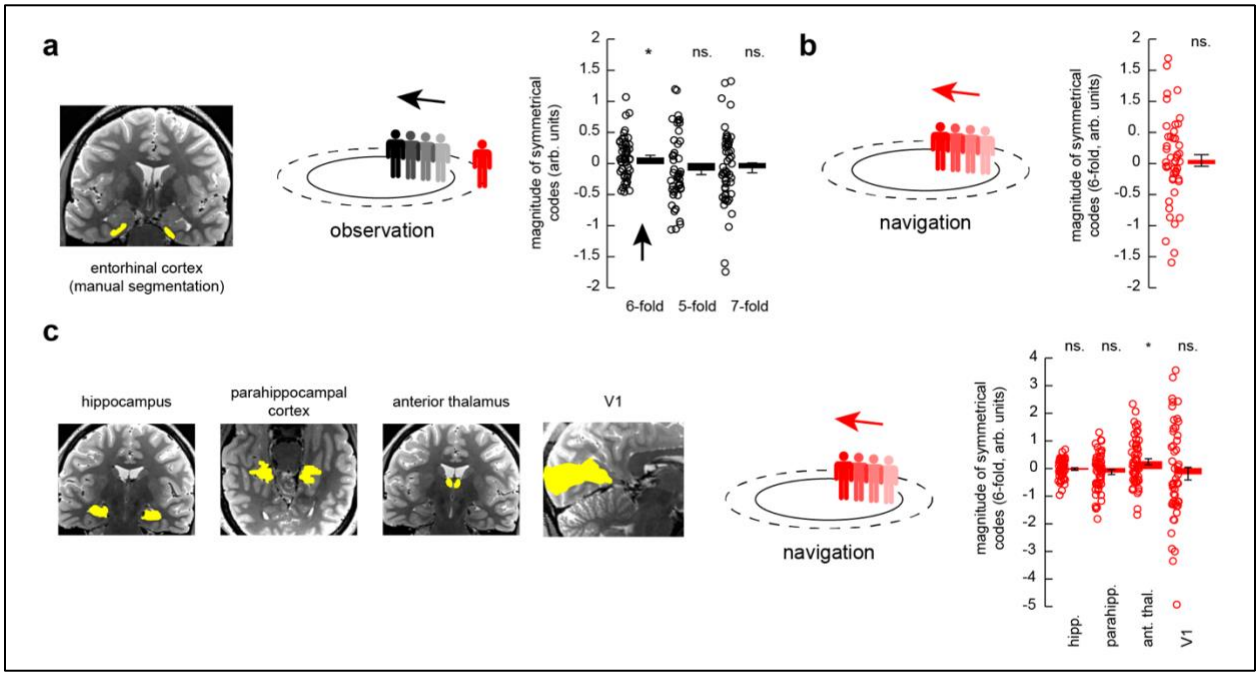

Fig. S3. Entorhinal grid-like codes during observation but not during navigation. (a) Results when manually segmenting the entorhinal cortex ROI. Magnitude of symmetrical codes (separate one-sample *t*-tests, one-tailed, multiple comparisons corrected; 6-fold: $N = 51$, $p = 0.032$; 5-fold: $N = 47$, $p = 0.102$; 7-fold: $N = 46$, $p = 0.178$) in the entorhinal cortex during observation periods (arbitrary units, arb. units). **(b)** No significant entorhinal grid-like codes during navigation periods (one-sample *t*-test, one-tailed, $N = 45$, $p = 0.323$; again, manual delineation of ROI), and **(c)** no significant grid-like codes in most of the control ROIs (separate one-sample *t*-tests, one-tailed, multiple comparisons corrected; hippocampus: $N = 52$, $p = 0.34$; parahippocampal cortex: $N = 56$, $p = 0.087$; anterior thalamus: $N = 54$, $p = 0.016$; V1: $N = 52$, $p = 0.222$). However, grid magnitudes were significantly increased in the anterior thalamus. * $p < 0.05$; error bars reflect the standard error of the mean, s.e.m. Source data are provided as a Source Data file.

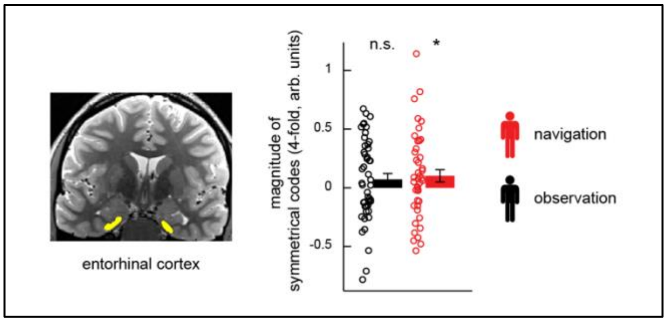

Fig. S4. Results of the 4-fold modulation analysis. No significant 4-fold modulation of the entorhinal cortex signal (automatic segmentation with ASHS) during observation (one-sample *t*-test, one-tailed, $N = 47$, $p = 0.082$; n.s., not significant) but significantly increased signal during navigation (one-sample *t*-test, one-tailed, $N = 43$, $p = 0.037$; *, significant). Error bars reflect the standard error of the mean, s.e.m. Source data are provided as a Source Data file.

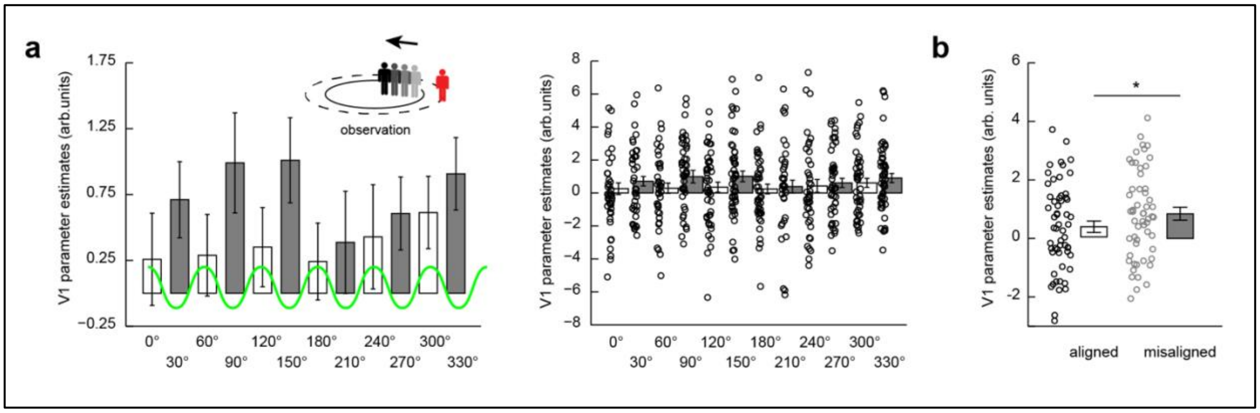

Fig. S5. V1 activation during aligned and misaligned paths observed. (a) *Left side:* V1 activation levels (quantified by parameter estimates, arbitrary units, arb. units) when observing aligned (0 modulo 60°) and misaligned (0 modulo 30°, indicated in bold) directions, sorted according to the putative grid orientation (0°) and obtained using 12-fold cross-validation (N = 58). The green line indicates hypothetical signal modulation as would be the case when assuming increased grid-like codes when observing/walking aligned (0 modulo 60°) with the internal grid orientation. *Right side*: Same data as left graph but with individual data points highlighted. **(b)** Average V1 activation levels for aligned and misaligned directions observed (paired-sample t-test, two-tailed, N = 58, p = 0.016). Error bars reflect the standard error of the mean, s.e.m; *, p < 0.05. Source data are provided as a Source Data file.

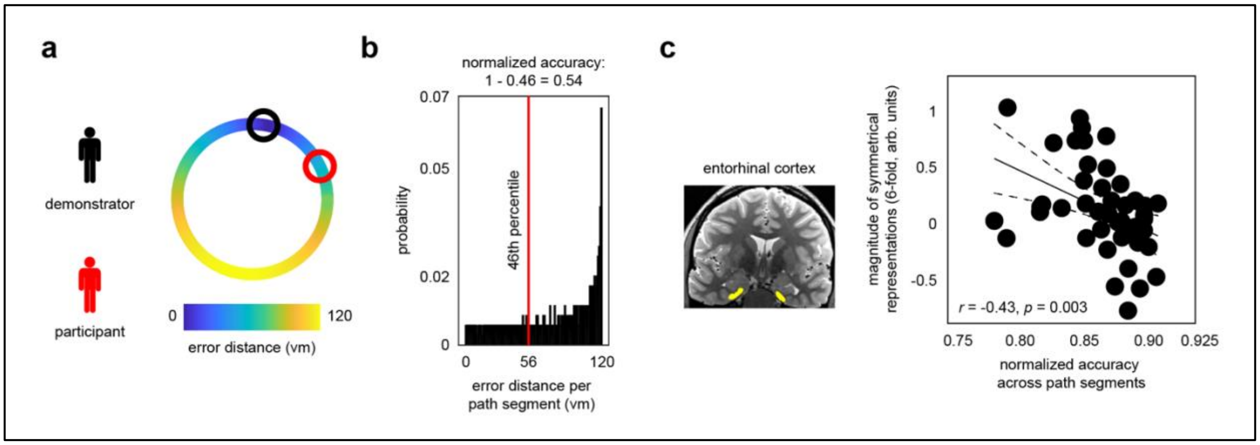

Fig. S6. Normalized accuracy measure and correlation with entorhinal grid-like codes during observation. (a) Color gradient indicates chance level at different points on the circumference of the movement area in virtual reality space (120 vm = maximum error distance). Black and red circles indicate exemplary endpoints of the demonstrator and participant, respectively, yielding an error distance of 46 vm for this specific path segment. **(b)** Probability distribution of all possible error distances. The normalized accuracy is calculated as the percentile rank of the actual path segment (i.e., endpoint that participant actually navigated towards) relative to all possible error distances (1-percentile/100). **(c)** Initial results remained virtually unchanged, once more showing increased grid-like codes in the entorhinal cortex during observation at lower overall accuracy (Pearson correlation, two-tailed, N = 47, p = 0.003). Source data are provided as a Source Data file.

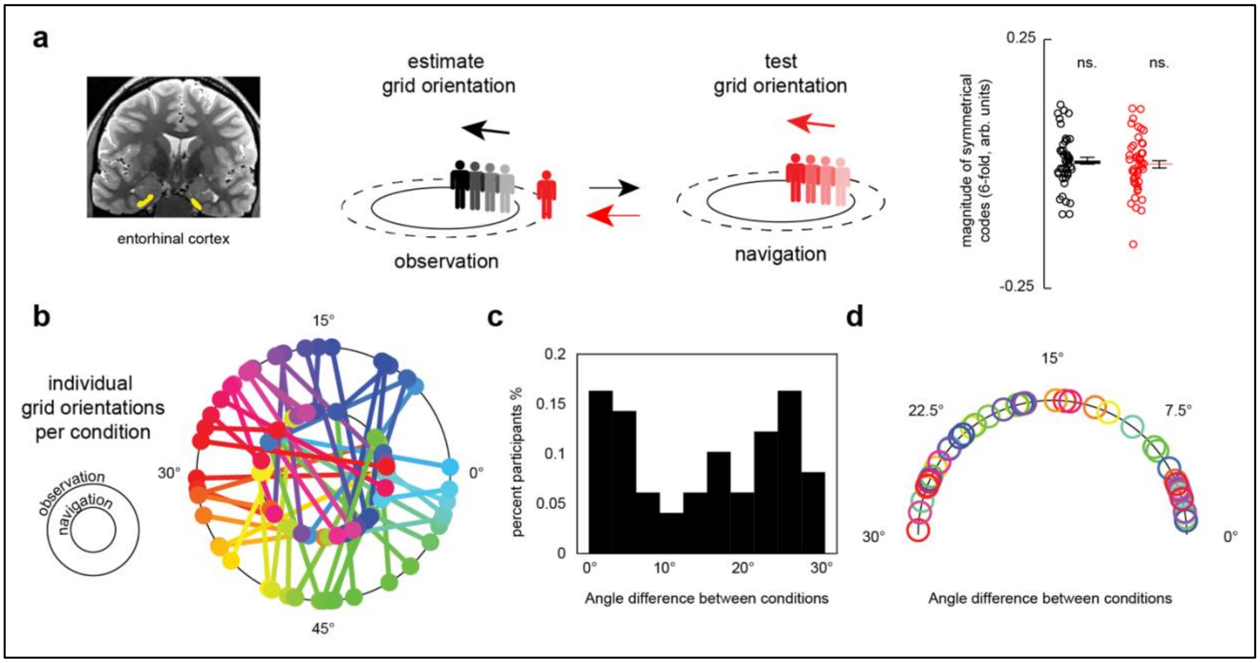

Fig. S7. Different grid orientations during observation and navigation in the entorhinal cortex. (a) No significant grid-like codes when estimating grid orientations on observation and testing them on navigation periods (and vice versa; separate one-sample *t*-tests, one-tailed, observation-to-navigation: N = 45, p = 0.206; navigation-to-observation: N = 45, p = 0.46). Error bars reflect the standard error of the mean, s.e.m. **(b)** Individual grid orientations plotted for observation (outer ring) and navigation (inner ring) conditions. Color variations indicate the different participants. **(c)** Distribution of differences in grid orientation angles between observation/navigation across the sample. **(d)** Differences in grid orientation angles between observation/navigation visualized once more. Data matches the distribution in (b), color values match those in (a). Source data are provided as a Source Data file.

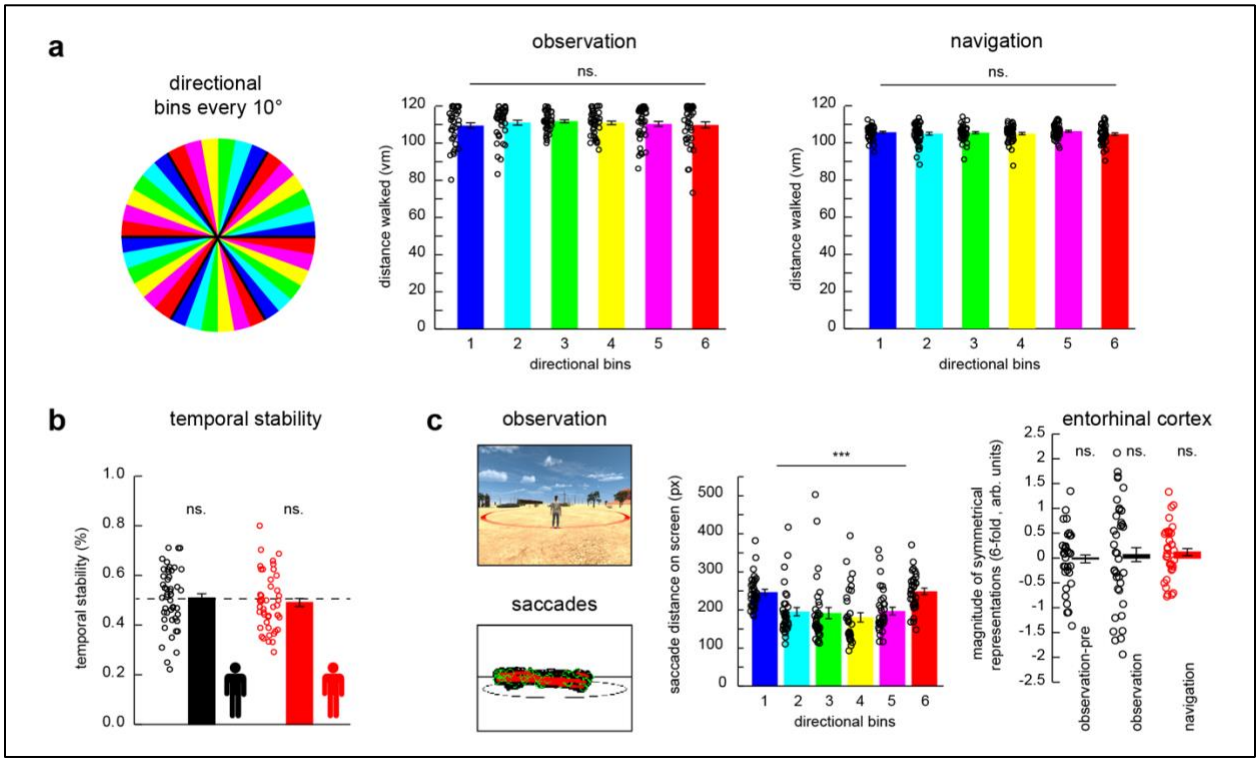

Fig. S8. Control analyses. (a, left panel) Unbiased estimation of grid orientations. We designed our task such that path segments were oriented along 36 directions that could be divided into 6 directional bins, spanning the 360°-VR-space with 10° angular resolution. (A, middle panel) There was no significant difference in the distance walked (vm) across directional bins during observation periods [one-way ANOVA, N = 48 (individual data points indicate the trial-wise distance walked in each of the directional bins and in each of the four task runs, based on the two trial sequences randomly generated before the start of the experiment, **Methods**), no significant main effect of directional bin, $p_{\text{two-tailed}} = 0.896$]. **(a, right panel)** This was also not the case during navigation periods [repeated measures ANOVA, N = 45 (same participant sample as during initial grid code analysis), no significant main effect of directional bin, $p_{\text{two-tailed}} = 0.968$, individual data points indicate the participant-specific distance walked in each of the directional bins, averaged across trials and runs]. **(b)** Temporal stability of grid-like coding was not significantly different between the conditions [paired-sample *t*-test, N = 44 (participant sample from which both observation- and navigation-based grid values were available), $p_{\text{two-tailed}} = 0.252$] but was generally low (i.e., not significantly different from the temporal stability threshold of 50% for observation/navigation periods marked with dashed line; separate one-sample *t*-tests, N = 44, $p_{\text{two-tailed}}$ = 0.723/0.36), indicating variability of both observation- and navigation-based grid orientations over time. **(c)** No effects of eye movements on entorhinal grid-like coding. There was a significant difference in the saccade distance on the screen across the six directional bins [repeated-measures ANOVA, N = 33, *** $p_{\text{two-tailed}} < 0.0001$, individual data points indicate the segment-specific saccade distance on screen (pixels, px) in each of the directional bins, averaged across trials and runs, see results above]. We thus repeated the analysis and randomly selected saccades to match the average, individual saccade distance along directional bins 2 to 5 (separate one-sample t-tests, one-tailed; initial analyses based on all saccades marked as "observation-pre": N = 33, p = 0.433; observation: N = 37, p = 0.35; navigation: N = 35, p = 0.096). Error bars reflect the standard error of the mean, s.e.m; ns., not significant. Source data are provided as a Source Data file.

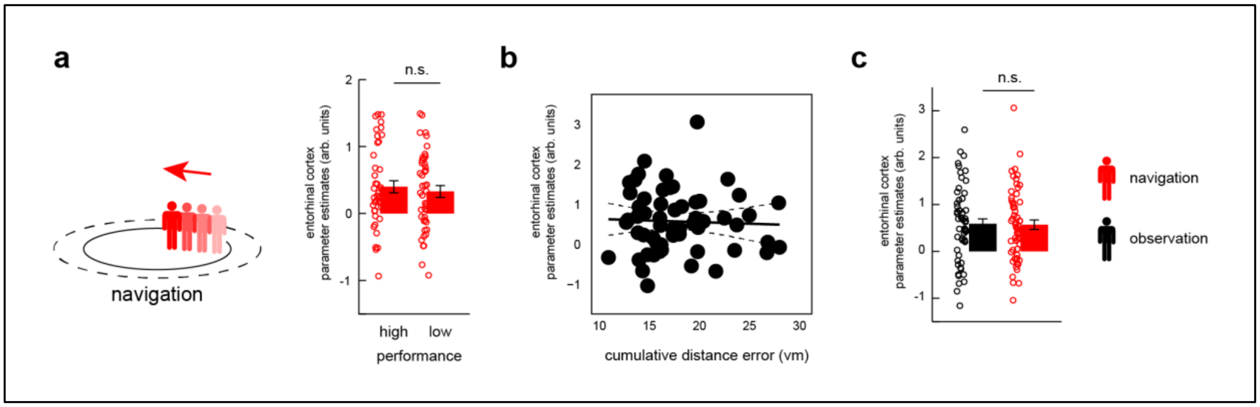

Fig. S9. Neural adaption analyses. (a) Activation levels (quantified as parameter estimates, arbitrary units, arb. units) extracted from the entorhinal cortex (automatically segmented using ASHS) during high- and low-performance navigation (<= 20 vm or > 20 vm cumulative distance error, respectively; paired-sample *t*-test, two-tailed, N = 44, p = 0.456). **(b)** No significant association between the average activation levels in the entorhinal cortex during navigation and the cumulative distance error (vm) across participants (Pearson correlation, two-tailed, N = 58, p = 0.755). **(c)** Activation levels in the entorhinal cortex during observation and navigation (paired-sample *t*-test, two-tailed, N = 58, p = 0.831). Error bars reflect the standard error of the mean, s.e.m; n.s., not significant. Source data are provided as a Source Data file.

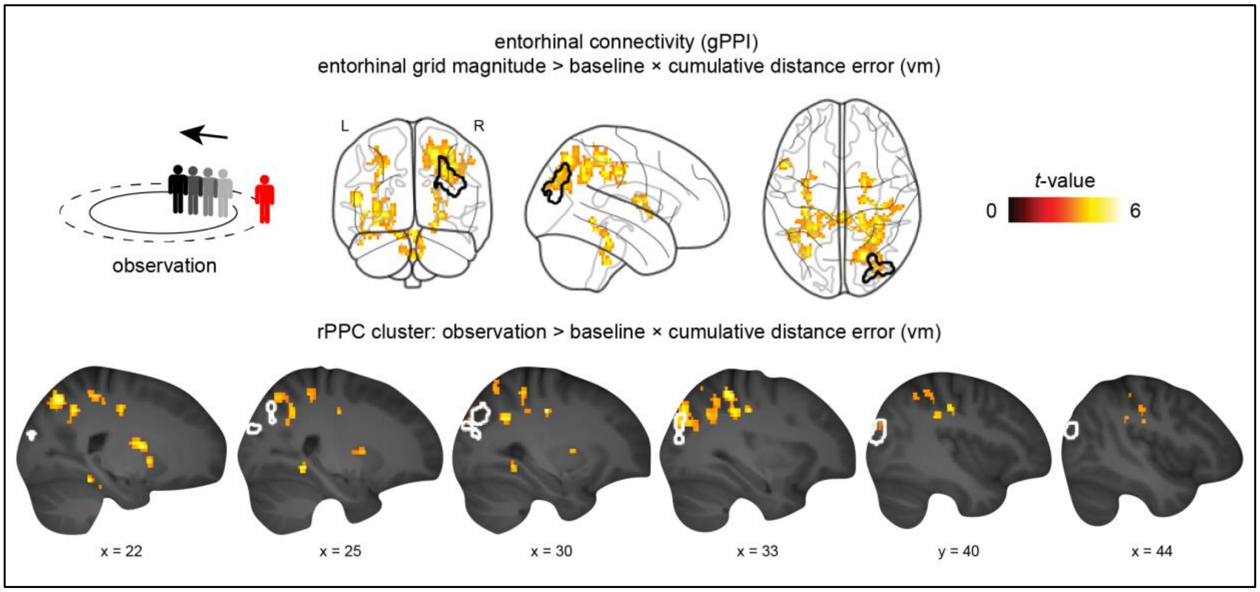

Fig. S10. Right posterior parietal cortex contributions during observation. Spatial overlap in the right posterior parietal cortex (rPPC) between entorhinal connectivity × performance results (i.e., entorhinal-cortical coupling during observation, time-locked to grid-like codes and negative association with performance; indicated with warm colors; taken from **Fig. 4d**) and activation × performance results (i.e., univariate activity, independent of grid-like codes, and positive association with performance; indicated through black outline; taken from **Fig. 2c**). Source data are provided as a Source Data file.

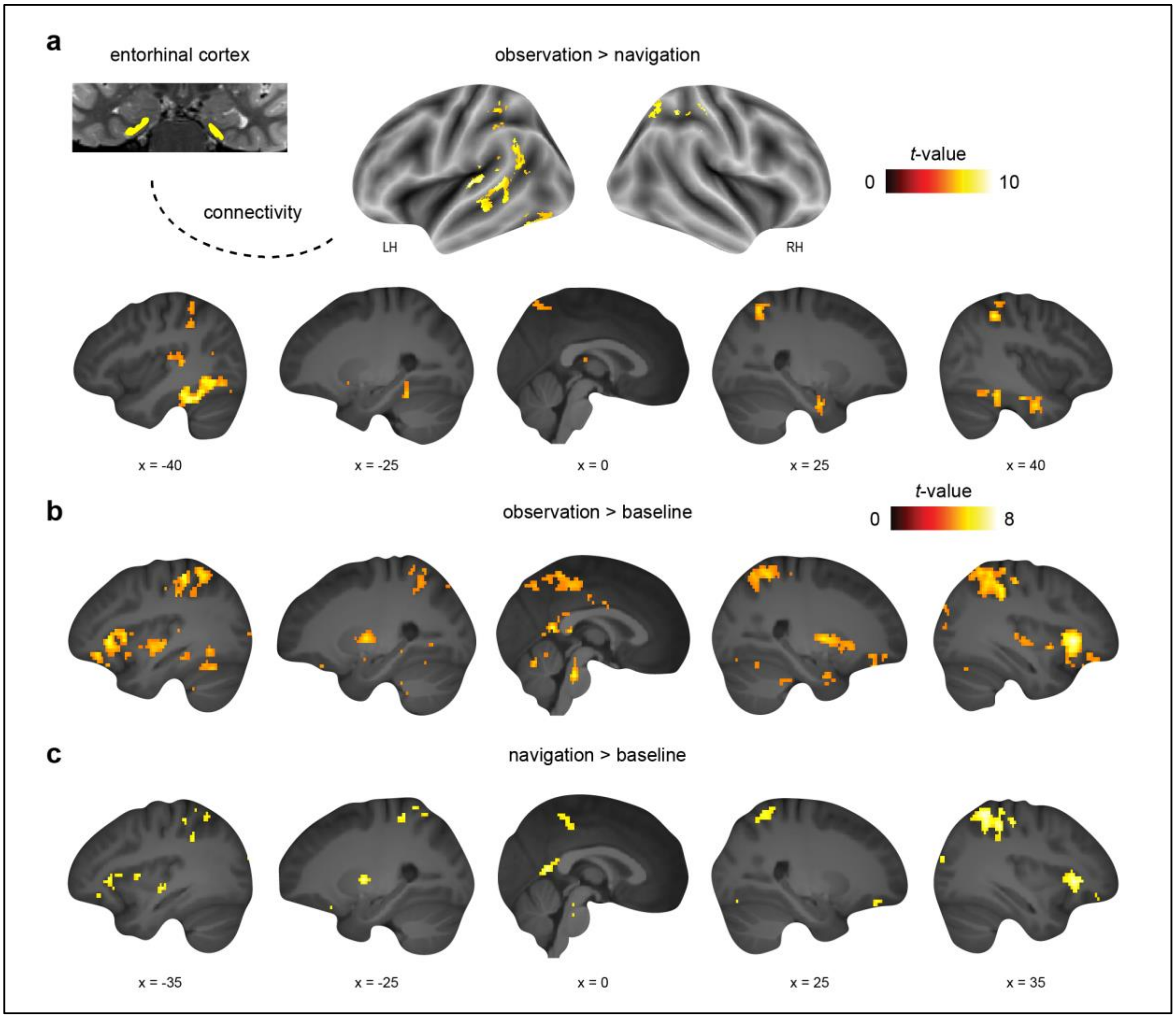

Fig. S11. Entorhinal-cortical connectivity changes independent of fluctuations in grid magnitude and performance. (a) Increased connectivity during observation compared to navigation. Results are shown at $p < 0.05$ FWE-corrected at cluster level (cluster-defining threshold of $p < 0.001$). **(b-c)** Connectivity changes during observation/navigation compared to the implicit (fixation) baseline. Please note that these results were thresholded at $p < 0.05$ FWE-corrected (i.e., without applying a cluster-defining threshold; tested with separate one-sample t-tests, $N = 47$). Source data are provided as a Source Data file.

Supplementary Tables

General note regarding tables displaying fMRI results

Unless stated otherwise, significance for all MRI analyses was assessed using cluster-inference with a cluster-defining threshold of $p < 0.001$ and a cluster-probability of $p < 0.05$ family-wise error (FWE) corrected for multiple comparisons. The corrected cluster size (i.e., the spatial extent of a cluster that is required in order to be labeled as significant) was calculated using the SPM extension "CorrClusTh.m" and the Newton-Raphson search method (script provided by Thomas Nichols, University of Warwick, United Kingdom, and Marko Wilke, University of Tübingen, Germany)[2].

MNI coordinates represent the location of cluster peak voxels. We report the first local maximum within each cluster. Complete tables (i.e., also providing the different local maxima within each cluster, reflecting the original output from the abovementioned script to calculate the corrected cluster size) are provided on the OSF[3]. Anatomical nomenclature for all tables was obtained from the Laboratory for Neuro Imaging (LONI) Brain Atlas (LBPA40, http://www.loni.usc.edu/atlases/)[14]. L, left; R, right; LH, left hemisphere; RH, right hemisphere.

[2] http://www2.warwick.ac.uk/fac/sci/statistics/staff/academic-research/nichols/scripts/spm/

[3] https://osf.io/mhtgp/

Table S1. Brain activation profiles during observation and navigation

Analysis consisted of separate one-sample *t*-tests (all *N* = 58), contrasts: observation > navigation (critical cluster size: 91 voxels), navigation > observation (91 voxels), observation > baseline (80 voxels), navigation > baseline (77 voxels).

	MNI				
Contrast & brain region	x	y	z	*Z* value	Cluster size
Observation > navigation:					
R middle occipital gyrus	44	-82	-6	Inf	7018
L superior temporal gyrus	-48	-42	6	Inf	7921
R postcentral gyrus	36	-18	24	7.62	1015
R middle frontal gyrus	18	38	0	7.51	3045
L precentral gyrus	-44	-10	39	6.75	853
L precuneus	-2	-60	45	5.97	296
L inferior frontal gyrus	-56	26	0	4.98	255
Navigation > observation:					
R superior occipital gyrus	18	-88	21	Inf	39636
R supramarginal gyrus	56	-36	39	Inf	1810
R middle temporal gyrus	52	-30	-9	4.49	333
Observation > baseline:					
R middle occipital gyrus	44	-80	-6	Inf	33139
L postcentral gyrus	-64	-6	30	7.08	625
R postcentral gyrus	68	-4	24	5.97	642
L postcentral gyrus	-6	-40	66	5.53	361
R insular cortex	34	-18	18	5.2	85
Navigation > baseline:					
L middle occipital gyrus	-26	-90	18	Inf	49749
L middle temporal gyrus	-58	6	-24	4.82	158

Table S2: Grid-like codes during observation (results based on the full sample)

Results (grid magnitudes in arbitrary units, arb. units) of the main grid code analysis based on two entorhinal cortex ROIs (defined through ASHS and manually segmented using ITK-SNAP) and four control ROIs (hippocampus, parahippocampal cortex, anterior thalamus, and primary visual cortex/V1). *N* describes the full participant sample available for the analyses (i.e., after applying restrictions for voxel selection but no outliers excluded, **Methods**). Analyses were performed using two complementary approaches (1: initial analysis using a one-sample *t*-test based on the full data set, no formal outliers excluded, 2: robust analysis using the Wilcoxon signed-rank test based on the full data set, no formal outliers excluded). Results were corrected for multiple comparisons using Bonferroni-correction ($\alpha_{Bonferroni}$ = 0.05/6 ROIs = 0.008). * $p < 0.001$, (*) $p < 0.05$ but not surviving Bonferroni-correction.

Grid-like codes during observation (analysis type)	*N*	Grid magnitude (mean ± s.e.m.)	Grid magnitude (median)	*t*-statistic	*p*-value (one-tailed)	*V*-statistic	Wilcoxon *p*-value
Entorhinal cortex (ASHS), 6-fold rotational symmetry	49	0.157 ± 0.06	0.125	*t*(48) = 2.45	0.009 (*)	868	0.005 *
Entorhinal cortex (ASHS), 5-fold rotational symmetry	49				0.146		0.248
Entorhinal cortex (ASHS), 7-fold rotational symmetry	49				0.23		0.422
Entorhinal cortex (ITK-SNAP), 6-fold rotational symmetry	51	0.088 ± 0.05	0.071	*t*(50) = 1.9	0.032 (*)	828	0.06 (~)
Entorhinal cortex (ITK-SNAP), 5-fold rotational symmetry	51				0.293		0.866
Entorhinal cortex (ITK-SNAP), 7-fold rotational symmetry	51				0.103		0.866
Hippocampus, 6-fold rotational symmetry	58				0.283		0.628
Parahippocampal cortex, 6-fold rotational symmetry	58				0.196		0.583
Anterior thalamus, 6-fold rotational symmetry	58				0.241		0.818
Primary visual cortex / V1, 6-fold rotational symmetry	58	-0.76 ± 0.17	-0.639	*t*(57) = -4.462	< 0.0001 *	371	< 0.0001 *

Table S3: Grid-like codes during navigation (results based on the full sample)

Results (grid magnitudes in arbitrary units, arb. units) of the main grid code analysis based on two entorhinal cortex ROIs (defined through ASHS and manually segmented using ITK-SNAP) and four control ROIs (hippocampus, parahippocampal cortex, anterior thalamus, and primary visual cortex/V1). *N* describes the full participant sample available for the analyses (i.e., after applying restrictions for voxel selection but no outliers excluded, **Methods**). Analyses were performed using two complementary approaches (1: initial analysis using a one-sample *t*-test based on the full data set, no formal outliers excluded, 2: robust analysis using the Wilcoxon signed-rank test based on the full data set, no formal outliers excluded). Results were corrected for multiple comparisons using Bonferroni-correction ($\alpha_{Bonferroni}$ = 0.05/6 ROIs = 0.008). (*) $p < 0.05$ but not surviving Bonferroni-correction, (~) tendency for $p < 0.05$.

Grid-like codes during navigation (analysis type)	*N*	Grid magnitude (mean ± s.e.m.)	Grid magnitude (median)	*t*-statistic	*p*-value (one-tailed)	*V*-statistic	Wilcoxon *p*-value
Entorhinal cortex (ASHS), 6-fold rotational symmetry	49	0.454 ± 0.22	0.230	$t(48) = 1.96$	0.028 (*)	796	0.034 (*)
Entorhinal cortex (ASHS), 5-fold rotational symmetry	49				0.362		0.559
Entorhinal cortex (ASHS), 7-fold rotational symmetry	49				0.218		0.336
Entorhinal cortex (ITK-SNAP), 6-fold rotational symmetry	51				0.191		0.487
Entorhinal cortex (ITK-SNAP), 5-fold rotational symmetry	51				0.068 (~)		0.865
Entorhinal cortex (ITK-SNAP), 7-fold rotational symmetry	51				0.088		0.92
Hippocampus, 6-fold rotational symmetry	58				0.166		0.568
Parahippocampal cortex, 6-fold rotational symmetry	58				0.162		0.818
Anterior thalamus, 6-fold rotational symmetry	58	0.687 +- 0.57	0.123		0.118	1079	0.042 (*)
Primary visual cortex / V1, 6-fold rotational symmetry	58				0.118		0.852

Table S4: Entorhinal connectivity changes time-locked to grid-like codes during observation, and association with performance

Analysis consisted of separate one-sample *t*-tests (all *N* = 47, same participant sample as during initial grid code analysis), contrasts: entorhinal grid magnitude during observation > baseline × cumulative distance error across all paths added as a covariate of interest (critical cluster size: 61 voxels). The findings were specific to lower performance (there were no significant connectivity changes related to increased navigation performance) and to observation periods only (there were no significant effects during navigation).

Contrast & brain region	MNI			*Z* value	Cluster size
	x	y	z		
Entorhinal grid magnitude during observation > baseline × covariate cumulative distance error across all paths:					
R superior parietal gyrus	22	-68	51	4.77	613
L inferior frontal gyrus	-52	16	6	4.72	73
R postcentral gyrus	36	-24	39	4.61	131
L hippocampus	-32	-34	-9	4.54	197
L angular gyrus	-30	-66	36	4.34	84
Brainstem	2	-32	-24	4.31	154
R putamen	22	0	12	4.31	92
L middle temporal gyrus	-42	-48	-3	4.26	100
R lingual gyrus	26	-48	-6	4.19	64
R middle occipital gyrus	34	-76	39	4.09	156
L insular cortex	-34	-6	0	4.05	107
L superior parietal gyrus	-26	-48	39	3.98	83

Table S5: Entorhinal connectivity changes independent of grid-like codes or performance

Analysis consisted of separate one-sample *t*-tests (all $N = 47$, same participant sample as during initial grid analysis), contrasts: observation > navigation (64 voxels), observation > baseline, navigation > baseline (both thresholded using $p < 0.05$ FWE-corrected for multiple comparisons, we report the first 10 clusters). There were no significant results for the reverse contrast navigation > observation.

	MNI				
Contrast & brain region	x	y	z	*Z* value	Cluster size
Observation > navigation:					
L superior temporal gyrus	-44	-28	12	4.97	664
L fusiform gyrus	-40	-40	-24	4.68	556
Thalamus	4	-16	12	4.66	179
Cerebellum	36	-40	-30	4.61	154
Striatum	-22	18	12	4.48	169
R postcentral gyrus	50	-28	57	4.38	203
R inferior temporal gyrus	32	-6	-30	4.34	195
Brainstem	8	-30	-30	4.08	102
R precuneus	8	-56	57	4.05	501
L superior parietal gyrus	-32	-38	48	4.02	238
R precuneus	8	-56	15	3.75	87
Observation > baseline:					
R inferior frontal gyrus	36	26	0	7.3	8464
Brainstem	-8	-20	-3	6.98	264
Brainstem	8	-22	0	6.73	167
L parahippocampal gyrus	-20	-20	-30	6.69	40
L cingulate gyrus	-6	-44	9	6.48	367
R inferior occipital gyrus	50	-74	-12	6.32	258
Brainstem	-10	-30	-30	6.28	305
Cerebellum	-42	-64	-24	6.09	159
L fusiform gyrus	-30	-60	-9	6.09	83
L lateral orbitofrontal gyrus	-34	38	-12	5.77	137

Table continues on the next page.

Contrast & brain region	MNI			*Z* value	Cluster size
	x	y	z		
Navigation > baseline:					
L inferior frontal gyrus	-58	12	12	6.37	442
R superior parietal gyrus	30	-54	60	6.32	1329
L parahippocampal gyrus	-22	-24	-27	6.21	20
L supramarginal gyrus	-54	-30	48	6.2	1170
Thalamus	-16	-18	-3	6.17	85
R precentral gyrus	60	12	18	6.15	85
Brainstem	-10	-30	-33	6.09	62
R insular cortex	34	26	0	6.07	180
L cingulate cortex	-4	-44	15	5.91	99
L putamen	-26	-6	3	5.87	90

References

1. Alink, A., Walther, A., Krugliak, A., Bosch, J. J. F. van den & Kriegeskorte, N. Mind the drift - improving sensitivity to fMRI pattern information by accounting for temporal pattern drift. *bioRxiv* 032391 (2015) doi:10.1101/032391.
2. Radvansky, G. A. & Zacks, J. M. Event boundaries in memory and cognition. *Curr Opin Behav Sci* **17**, 133–140 (2017).
3. Brunec, I. K., Moscovitch, M. & Barense, M. D. Boundaries Shape Cognitive Representations of Spaces and Events. *Trends Cogn Sci* **22**, 637–650 (2018).
4. Doeller, C. F., Barry, C. & Burgess, N. Evidence for grid cells in a human memory network. *Nature* **463**, 657–661 (2010).
5. Kunz, L. *et al.* Reduced grid-cell-like representations in adults at genetic risk for Alzheimer's disease. *Science (1979)* **350**, 430–433 (2015).
6. Miller, J. *et al.* Lateralized hippocampal oscillations underlie distinct aspects of human spatial memory and navigation. *Nat Commun* **9**, 2423 (2018).
7. Horner, A. J., Bisby, J. A., Zotow, E., Bush, D. & Burgess, N. Grid-like Processing of Imagined Navigation. *Current Biology* **26**, 842–847 (2016).
8. Kunz, L. *et al.* Mesoscopic Neural Representations in Spatial Navigation. *Trends in Cognitive Sciences* vol. 23 615–630 Preprint at https://doi.org/10.1016/j.tics.2019.04.011 (2019).
9. Barron, H. C., Garvert, M. M. & Behrens, T. E. J. Repetition suppression: A means to index neural representations using BOLD? *Philosophical Transactions of the Royal Society B: Biological Sciences* vol. 371 Preprint at https://doi.org/10.1098/rstb.2015.0355 (2016).
10. Couey, J. J. *et al.* Recurrent inhibitory circuitry as a mechanism for grid formation. *Nature Neuroscience 2013 16:3* **16**, 318–324 (2013).
11. Long, X., Deng, B., Cai, J., Chen, Z. S. & Zhang, S.-J. A compact spatial map in V2 visual cortex. *bioRxiv* 2021.02.11.430687 (2021) doi:10.1101/2021.02.11.430687.
12. Maass, A., Berron, D., Libby, L. A., Ranganath, C. & Düzel, E. Functional subregions of the human entorhinal cortex. *Elife* **4**, 1–20 (2015).
13. Bellmund, J. L., Deuker, L., Navarro Schröder, T. & Doeller, C. F. Grid-cell representations in mental simulation. *Elife* **5**, (2016).
14. Shattuck, D. W. *et al.* Construction of a 3D probabilistic atlas of human cortical structures. *Neuroimage* **39**, 1064–1080 (2008).
