# Supplementary material for: Entorhinal grid-like codes and time-locked network dynamics track others navigating through space
Source: Nat Commun. 2023 Jan 31;14:231. doi: 10.1038/s41467-023-35819-3 (PMC9889810; doi:10.1038/s41467-023-35819-3)
Supplement: Supplementary file 3 — Reporting Summary [file 41467_2023_35819_MOESM3_ESM.pdf]

nature portfolio

Corresponding author(s): Isabella Wagner
Last updated by author(s): Dec 9, 2022

Reporting Summary

Nature Portfolio wishes to improve the reproducibility of the work that we publish. This form provides structure for consistency and transparency in reporting. For further information on Nature Portfolio policies, see our Editorial Policies and the Editorial Policy Checklist.

Statistics

For all statistical analyses, confirm that the following items are present in the figure legend, table legend, main text, or Methods section.

n/a	Confirmed	
☐	☒	The exact sample size (*n*) for each experimental group/condition, given as a discrete number and unit of measurement
☐	☒	A statement on whether measurements were taken from distinct samples or whether the same sample was measured repeatedly
☐	☒	The statistical test(s) used AND whether they are one- or two-sided<br>*Only common tests should be described solely by name; describe more complex techniques in the Methods section.*
☐	☒	A description of all covariates tested
☐	☒	A description of any assumptions or corrections, such as tests of normality and adjustment for multiple comparisons
☐	☒	A full description of the statistical parameters including central tendency (e.g. means) or other basic estimates (e.g. regression coefficient) AND variation (e.g. standard deviation) or associated estimates of uncertainty (e.g. confidence intervals)
☐	☒	For null hypothesis testing, the test statistic (e.g. *F*, *t*, *r*) with confidence intervals, effect sizes, degrees of freedom and *P* value noted<br>*Give P values as exact values whenever suitable.*
☒	☐	For Bayesian analysis, information on the choice of priors and Markov chain Monte Carlo settings
☒	☐	For hierarchical and complex designs, identification of the appropriate level for tests and full reporting of outcomes
☐	☒	Estimates of effect sizes (e.g. Cohen's *d*, Pearson's *r*), indicating how they were calculated

Our web collection on statistics for biologists contains articles on many of the points above.

Software and code

Policy information about availability of computer code

Data collection	All imaging data were collected at the Neuroimaging Center of the University of Vienna, using a 3T Skyra MR-scanner (Siemens, Erlangen, Germany) equipped with a 32-channel head coil. The task was implemented with Unity (software version 2019.4.5f1, https://unity.com).
Data analysis	Data was analyzed with openly available software code including SPM (software version 12; http://www.fil.ion.ucl.ac.uk/spm/), Functional Magnetic Resonance Imaging of the Brain (FMRIB) Software Library (FSL; software version 5.0.1; https://fsl.fmrib.ox.ac.uk/fsl), Grid Code Analysis Toolbox (GridCAT; software version 1.0.4; https://www.nitrc.org/projects/gridcat), Fieldtrip (latest software version downloaded on 4th March 2021, updated daily; https://www.fieldtriptoolbox.org), Automated Segmentation of the Hippocampal Subfields (software version 1.0.0, https://sites.google.com/site/hipposubfields/), ITK-SNAP (software version 3.6, www.itksnap.org), Matlab (The Mathworks, Natick, MA, USA; software version R2019b), R (software version 4.0.5.; https://www.r-project.org), R-package "emmeans" (software version 1.5.5.; https://cran.r-project.org/web/packages/emmeans/index.html). All custom code is provided on the Open Science Framework (https://osf.io/mhtgp/).

For manuscripts utilizing custom algorithms or software that are central to the research but not yet described in published literature, software must be made available to editors and reviewers. We strongly encourage code deposition in a community repository (e.g. GitHub). See the Nature Portfolio guidelines for submitting code & software for further information.

Data

Policy information about availability of data

All manuscripts must include a data availability statement. This statement should provide the following information, where applicable:

- Accession codes, unique identifiers, or web links for publicly available datasets
- A description of any restrictions on data availability
- For clinical datasets or third party data, please ensure that the statement adheres to our policy

Raw, anonymized fMRI data are available upon request to the corresponding author (isabella.wagner@univie.ac.at). At present, participant informed consent does not allow for depositing the full data set. All analyses are based on openly available software. Source data to reproduce all graphs (behavioral performance and ROI-based results of all grid analyses), as well as (un-)thresholded statistical whole- brain fMRI maps and their corresponding results tables are openly available at the Open Science Framework (https://osf.io/mhtgp/).

Human research participants

Policy information about studies involving human research participants and Sex and Gender in Research.

Reporting on sex and gender	Persons of both sexes (female, male) participated in the study (45 females, 15 males). Biological sex was determined by self-report. Sex differences were not at the focus of this study and sex-based analyses were thus not performed. Thus, we propose that the findings of this study apply to both sexes.
Population characteristics	Sixty participants volunteered for this study (aged 18-29 years, mean = 22 years, 45 females, 7 left-handed). All participants were healthy, had normal or corrected-to-normal vision, gave written informed consent prior to participation, and received monetary compensation. Two participants were excluded from data analyses (one participant due to an anatomical brain abnormality, and one participant due to low performance in the socio-spatial navigation task), which left 58 participants for all following analyses (aged 18-28 years, mean = 22 years, 44 females, 7 left-handed).
Recruitment	Participants were recruited from the online participant recruitment system of the University of Vienna (https://labs-univie.sona-systems.com/Default.aspx?ReturnUrl=%2f). There was no self-selection bias in recruitment; participants were solely selected based on their ability to participate in the study (based on their health, normal or corrected-to-normal vision, eligibility for MRI scanning) and based on their ability to provide written informed consent.
Ethics oversight	The study was reviewed and ethically approved by the ethics committee of the University of Vienna (Vienna, Austria; reference number 00538).

Note that full information on the approval of the study protocol must also be provided in the manuscript.

Field-specific reporting

Please select the one below that is the best fit for your research. If you are not sure, read the appropriate sections before making your selection.

☒ Life sciences ☐ Behavioural & social sciences ☐ Ecological, evolutionary & environmental sciences

For a reference copy of the document with all sections, see nature.com/documents/nr-reporting-summary-flat.pdf

Life sciences study design

All studies must disclose on these points even when the disclosure is negative.

Sample size	Sample size calculations were based on fMRI studies that tested grid-like representations during self-related (mental) navigation (pooled effect size, Cohen's d=0.49; Doeller et al., 2010, Bellmund et al., 2016; Horner et al., 2016). Based on this medium sized effect, we would need 35 participants to detect grid-like representations during (self-related) navigation with a power of 80% (α=0.05). Since socio-spatial navigation has not been tested previously, we decided to increase the number to 60 participants (already accounting for potential dropouts).
Data exclusions	General sample:<br>Two participants were excluded from data analyses (one participant due to an anatomical brain abnormality, and one participant due to low performance in the socio-spatial navigation task), which left 58 participants for all following analyses (aged 18-28 years, mean = 22 years, 44 females, 7 left-handed).<br><br>Eye tracking:<br>Due to technical problems eye tracking was only possible in a subsample of 47 participants.<br><br>Grid analyses:<br>The entorhinal cortex lies in close proximity to the temporal horn of the lateral ventricle. Such tissue borders are often associated with lower signal-to-noise ratio, which is also what we experienced in a subsample of participants. To circumvent this issue, we only considered voxels that exceeded a signal-to-noise threshold of 0.8, leading to the fact that voxels along the anterior-medial entorhinal cortex border were partly

	dropped from the analyses (participants were excluded if there were less than 5 voxels left in the mask, and two participants were fully excluded from all grid code analyses involving the entorhinal cortex). After applying these restrictions, the final participant sample for which entorhinal cortex data was available comprised 49 (ASHS; 23 ± 13 voxels) or 51 (ITK-SNAP; 38 ± 25 voxels) participants. Additional analyses with the control ROIs included the full sample of 58 participants.
Replication	Grid-like codes were analyzed in ROIs based on automated segmentation and on manual delineation of the region (see below). Both types of analyses yielded the same results (significantly increased grid-like codes in the entorhinal cortex during observation periods). Thus, analysis using the manual delineation replicated the main analysis (using the automated segmentation).
Randomization	There were no experimental groups.
Blinding	There were no experimental groups, blinding of experimenters was therefore not necessary.

Reporting for specific materials, systems and methods

We require information from authors about some types of materials, experimental systems and methods used in many studies. Here, indicate whether each material, system or method listed is relevant to your study. If you are not sure if a list item applies to your research, read the appropriate section before selecting a response.

Materials & experimental systems

n/a	Involved in the study
☒	☐ Antibodies
☒	☐ Eukaryotic cell lines
☒	☐ Palaeontology and archaeology
☒	☐ Animals and other organisms
☒	☐ Clinical data
☒	☐ Dual use research of concern

Methods

n/a	Involved in the study
☒	☐ ChIP-seq
☒	☐ Flow cytometry
☐	☒ MRI-based neuroimaging

Magnetic resonance imaging

Experimental design

Design type	Task design, event-related.
Design specifications	Each participant underwent a single MRI session, starting out with the acquisition of the structural brain images while participants completed the task familiarization period (participants trained the subsequent socio-spatial navigation task during one run, all completed the same trials). This was followed by four runs of the actual socio-spatial navigation task and the acquisition of functional brain images. Each run consisted of 12 trials each (i.e., consisting of 12 observation and 12 navigation periods).
Behavioral performance measures	Behavioral performance was quantified as cumulative distance error in virtual meters (vm) indicating the deviation from the demonstrator's paths (mean ± standard error of the mean, s.e.m.; calculated as the average distance between the demonstrator's and the participant's endpoints across the three different segments of a given path).

Acquisition

Imaging type(s)	Functional and structural images.
Field strength	3 Tesla
Sequence & imaging parameters	All imaging data were collected at the Neuroimaging Center of the University of Vienna, using a 3T Skyra MR-scanner (Siemens, Erlangen, Germany) equipped with a 32-channel head coil. During each of the four task runs, we acquired on average 474 (± 7, s.d.) T2*-weighted blood oxygenation level-dependent (BOLD) images, using a partial-volume echo-planar imaging (EPI) sequence with the following parameters: repetition time (TR) = 2.029 s, echo time (TE) = 30 ms, number of slices = 30 axial slices, slice order = interleaved acquisition, field of view (FoV) = 216 mm, flip angle = 90°, slice thickness = 3mm, in-place resolution = 2 × 2 mm, using parallel imaging with a GRAPPA acceleration factor of 2. Slices were oriented parallel to the long axis of the hippocampus.<br><br>We additionally acquired 30 images for post-hoc artifact correction using the abovementioned functional sequence but reversing the phase-encoding direction, as well as 10 whole-brain EPI images with the following parameters: repetition time (TR) = 2.832 s, echo time (TE) = 30 ms, number of slices = 42 axial slices, slice order = interleaved acquisition, field of view (FoV) = 216 mm, flip angle = 90°, slice thickness = 3mm, in-place resolution = 2 × 2 mm, using parallel imaging with a GRAPPA acceleration factor of 2. Slices were oriented parallel to the long axis of the hippocampus.<br><br>The T1-weighted structural image was acquired using a Magnetization-Prepared Rapid Gradient Echo (MPRAGE) sequence with the following parameters: TR = 2.3 s; TE = 2.43 ms; FoV = 240 mm, flip angle = 8°, voxel size = 0.8 mm isotropic. To delineate the entorhinal cortex, we acquired a T2-weighted structural image using a turbo-spin-echo (TSE)

	Sampling Perfection with Application optimized Contrasts using different flip angle Evolution (SPACE) sequence with the following parameters: TR = 3.2 s; TE = 564 ms; FoV = 256 mm, voxel size = 0.8 mm isotropic. These slices were oriented perpendicular to the long axis of the hippocampus.
Area of acquisition	Whole-brain / partial volume focused on the hippocampus and entorhinal cortex; see above.

Diffusion MRI ☐ Used ☒ Not used

Preprocessing

Preprocessing software	The MRI data were processed using SPM12 (http://www.fil.ion.ucl.ac.uk/spm/) in combination with Matlab (The Mathworks, Natick, MA, USA; software version R2019b) and the Functional Magnetic Resonance Imaging of the Brain (FMRIB) Software Library (FSL, v5.0.1; https://fsl.fmrib.ox.ac.uk/fsl/fslwiki/; Jenkinson et al., 2012). The first six volumes were excluded to allow for T1-equilibration. The remaining volumes were slice-time-corrected to the middle slice and realigned to the mean image calculated across the four task runs. Potential image distortions were corrected by applying FSL's "topup" command: we calculated the mean image based on the additional volumes acquired (phase-encoding direction reversed). Together with the original fMRI data, this image was then used to estimate and correct susceptibility-induced distortions. Since grid-like codes were analyzed in the participant-specific image space, we refrained from normalizing the data but applied a 3D Gaussian smoothing kernel (5 mm full-width at half maximum, FWHM).<br><br>For the whole-brain group analyses, the distortion-corrected data was additionally normalized into standard space. The structural scan was co-registered to the mean functional image and segmented into grey matter, white matter, and cerebrospinal fluid using the "New Segmentation" algorithm. All images (functional and structural) were spatially normalized to the Montreal Neurological Institute (MNI) EPI template using Diffeomorphic Anatomical Registration Through Exponentiated Lie Algebra (DARTEL, Ashburner, 2007), and functional images were smoothed with a 3D Gaussian kernel (5 mm FWHM).
Normalization	All images (functional and structural) were spatially normalized to the Montreal Neurological Institute (MNI) EPI template (MNI-152) using Diffeomorphic Anatomical Registration Through Exponentiated Lie Algebra (DARTEL, Ashburner, 2007).
Normalization template	MNI-152
Noise and artifact removal	Volumes were realigned to the mean image calculated across the four task runs. Potential image distortions were corrected by applying FSL's "topup" command: we calculated the mean image based on the additional volumes acquired (phase-encoding direction reversed). Together with the original fMRI data, this image was then used to estimate and correct susceptibility-induced distortions.
Volume censoring	No volumes were censored.

Statistical modeling & inference

Model type and settings	A mass univariate approach was used.<br><br>Using a Generalized Linear Modeling (GLM), the BOLD response during the socio-spatial navigation task was modeled using separate task regressors, time-locked to the onsets of the respective events (cues, observation periods, navigation periods, feedback). All events were estimated as boxcar functions of specific durations and were convolved with the SPM default canonical hemodynamic response function (HRF). Cue and Feedback periods were modelled with a duration of 2 and 1 seconds, respectively. The duration of observation and navigation periods varied depending on the path length and the participant's behavior, and was defined through the on- and offsets of the VR environment on the computer screen (ranging between 18-40 seconds; see above). This included events such as orientation adjustments (rotations), walked path segments (translation periods), and time periods during which no movement occurred (short standing periods in-between). To account for noise due to head movement, we included the six realignment parameters, their first derivatives, and the squared first derivatives into the design matrix. A high-pass filter with a cutoff at 128 s was applied. The four runs of the socio-spatial navigation task were combined into one first-level model and contrasts were created ([observation ∩ navigation] > implicit baseline, observation/navigation > implicit baseline, observation > navigation and vice versa), collapsing across the different runs. To test for group effects, these contrast images were submitted to one-sample t-tests.<br><br>Additionally, we were interested in whether activation changes during observation and/or navigation scaled with individual socio-spatial navigation performance. We thus ran two linear regression analyses (contrasts observation/navigation > implicit baseline) and added the cumulative distance error (vm, obtained during navigation periods and averaged across all three points of a path trajectory) as a covariate of interest.
Effect(s) tested	Contrasts were created ([observation ∩ navigation] > implicit baseline, observation/navigation > implicit baseline, observation > navigation and vice versa), collapsing across the different runs. To test for group effects, these contrast images were submitted to one-sample t-tests.

Specify type of analysis: ☐ Whole brain ☐ ROI-based ☒ Both

Entorhinal cortex:<br>We used the T2-weighted structural scans to anatomically delineate individual entorhinal cortices. First, ROIs were automatically generated using Automated Segmentation of the Hippocampal Subfields (ASHS, https://sites.google.com/site/hipposubfields/; Yushkevich et al., 2015). Second, to verify the ASHS-based segmentation, we also performed manual delineation of the entorhinal cortex by tracing its anatomical borders on the structural image. This was done using ITK-SNAP (version 3.6.; www.itksnap.org;

Anatomical location(s)	Yushkevich et al., 2006), following the segmentation protocol provided by Berron and colleagues (Berron et al., 2017).<br><br>As we did not have a specific hypothesis regarding the laterality of brain effects, we collapsed the left and right masks into a bilateral entorhinal cortex image (for both the ASHS- and the ITK-SNAP-based delineations). These masks were then binarized and transformed into the participant-specific space of the functional images. Since the functional images were only partial-volume slabs, co-registration was aided by an additional intermediate step that involved the mean whole-brain functional image (Stangl et al., 2018). First, each participant's T2-weighted structural image (together with the individual entorhinal mask) was co-registered to match the orientation of the mean whole-brain functional image. Second, the mean whole-brain functional image (together with the co-registered individual entorhinal mask) was co-registered to match the orientation of the mean partial-volume functional image (mean ± s.d.; ASHS, 56 ± 13 voxels; ITK-SNAP, 104 ± 23 voxels).<br><br>The quality of co-registration was confirmed through visual inspection of each mask's overlap with the individual (co-registered) structural and functional data. The entorhinal cortex lies in close proximity to the temporal horn of the lateral ventricle. Such tissue borders are often associated with lower signal-to-noise ratio, which is also what we experienced in a subsample of participants. To circumvent this issue, we only considered voxels that exceeded a signal-to-noise threshold of 0.8, leading to the fact that voxels along the anterior-medial entorhinal cortex border were partly dropped from the analyses (participants were excluded if there were less than 5 voxels left in the mask, and two participants were fully excluded from all grid code analyses involving the entorhinal cortex). After applying these restrictions, the final participant sample for which entorhinal cortex data was available comprised 49 (ASHS; 23 ± 13 voxels) or 51 (ITK-SNAP; 38 ± 25 voxels) participants.<br><br>Control ROIs:<br>To test whether grid-like codes were also detectable in other regions, we chose several control ROIs known to be involved in spatial navigation and visual processing but for which no significant grid-like coding was reported so far. These included the adjacent hippocampus, the parahippocampal cortex, the anterior thalamus, and the primary visual cortex (V1). Both the hippocampus and parahippocampal cortex masks were defined using the ASHS algorithm (the hippocampus was defined by merging the hippocampal subfields cornu ammonis (CA) 1-4 and the subiculum). To delineate the anterior thalamus, we used the stereotactic mean anatomical atlas provided by Krauth and colleagues (Krauth et al., 2010) (© University of Zurich and ETH Zurich, Axel Krauth, Rémi Blanc, Alejandra Poveda, Daniel Jeanmonod, Anne Morel, Gábor Székely), which is based on histological, cytoarchitectural features defined ex vivo (Morel, 2007). We specified the anterior thalamus by combining the anterior dorsal, -medial, and -ventral nucleus masks. The V1 mask was created using the Automatic Anatomical Labeling (AAL) atlas (Tzourio-Mazoyer et al., 2002).<br><br>As above, left and right masks were combined into bilateral volumes and were transformed into the participant-specific image space (hippocampus, 447 ± 56 voxels; parahippocampal cortex, 160 ± 26 voxels; anterior thalamus, 41 ± 6 voxels; V1, 1136 ± 297 voxels). The quality of co-registration was confirmed through visual inspection of each mask's overlap with the individual structural and functional data of each participant (final sample, N = 58 participants).
Statistic type for inference<br>(See Eklund et al. 2016)	Unless stated otherwise, significance for all whole-brain fMRI analyses was assessed using cluster-inference with a cluster-defining threshold of $p < 0.001$ and a cluster-probability of $p < 0.05$ family-wise error (FWE) corrected for multiple comparisons. The corrected cluster size (i.e., the spatial extent of a cluster that is required in order to be labeled as significant) was calculated using the SPM extension "CorrClusTh.m" and the Newton-Raphson search method (script provided by Thomas Nichols, University of Warwick, United Kingdom, and Marko Wilke, University of Tübingen, Germany; http://www2.warwick.ac.uk/fac/sci/statistics/staff/academic-research/nichols/scripts/spm/). Anatomical nomenclature for all tables was obtained from the Laboratory for Neuro Imaging (LONI) Brain Atlas (LBPA40, http://www.loni.usc.edu/atlases/).
Correction	Family-wise error (FWE) correction for multiple comparisons.

Models & analysis

n/a	Involved in the study
☐	☒ Functional and/or effective connectivity
☒	☐ Graph analysis
☒	☐ Multivariate modeling or predictive analysis

Functional and/or effective connectivity	We performed generalized psychophysiological interaction analysis (gPPI; McLaren et al., 2012). We took the anatomical boundaries of the bilateral posterior-medial entorhinal cortex as a seed (Maass et al., 2015), extracted the first eigenvariate of its functional timecourse and adjusted for average activation levels using an F-contrast. The timecourse was then deconvolved to estimate the putative neural activity of the seed region (i.e., the physiological factor) and was multiplied with boxcar functions that defined the specific task events (i.e., the psychological factor). The resulting vectors were convolved with the canonical HRF, yielding one psychophysiological interaction regressor per condition-of-interest (i.e., for parametric modulation regressors that captured fluctuations in entorhinal grid magnitude during observation/navigation periods), and were contrasted against the implicit baseline.<br><br>Group-level connectivity analyses were performed using a set of one-sample t-tests. Again, linear regression

analysis was used to test for connectivity changes time-locked to fluctuations in entorhinal grid magnitude that potentially scaled with socio-spatial navigation performance by adding the cumulative distance error as a covariate of interest.
